# The Relationship Analysis on Corn Stalk Rot and Ear Rot According to *Fusarium* Species and Fumonisin Contamination in Kernels

**DOI:** 10.3390/toxins11060320

**Published:** 2019-06-05

**Authors:** Lina Li, Qing Qu, Zhiyan Cao, Zhengyu Guo, Hui Jia, Ning Liu, Yanhui Wang, Jingao Dong

**Affiliations:** 1 Plant Pathogenic Mycotoxin and Molecular Plant Pathology Laboratory of Hebei Agricultural University, Hebei Key Laboratory of Plant Physiology and Molecular Pathology, Hebei Agricultural University, Baoding 071001, China; lenali0304@126.com (L.L.); qu_qing@126.com (Q.Q.); hui_jiahui@126.com (H.J.); lning121@126.com (N.L.); 2 Maize Research Institute to Shanxi Academy of Agriculture Sciences, Xinzhou 034000, China; ymsgzy@163.com

**Keywords:** corn stalk rot, *Fusarium* species, ear rot, fumonisins

## Abstract

*Fusarium* diseases, including corn root rot, sheath rot, stalk rot, and ear rot are frequently occurring in maize producing areas of China. *Fusarium* stalk rot and ear rot are the most serious diseases and often occur at the same time, but it is unclear whether there is a correlation between *Fusarium* composition and disease occurrence. This study was conducted to clarify the relationship between the two diseases. A total of 49 corn stalk rot samples were collected from 15 regions of eight provinces in China from 2016 to 2018. The pathogens were isolated and identified separately from stalks, ear stems, and kernels. The contents of the fumonisins (FB_1_ and FB_2_) were detected in kernels. The results showed that the main *Fusarium* species were found in corn kernels, ear stems and stalks at the same time. The results showed that 1201 strains of *Fusarium verticillioides*, 668 strains of *Fusarium oxysporum*, 574 strains of *Fusarium graminearum* species complex (FGSC), 318 strains of *Fusarium equiseti*, 95 strains of *Fusarium proliferatum*, and 40 strains of *Fusarium subglutinans* were isolated from 1470 corn kernels, 245 ear stems, and 1225 stalks randomly selected from 49 samples. The contamination rate of fumonisins in the 49 samples was 57.1% with an average content of 1.9 μg/g, of which four samples exhibited higher levels as set by the European Commission (4.0 μg/g). These results provide a certain association between stalk rot and ear rot and lay a foundation to study the relationships among *Fusarium* maize diseases.

## 1. Introduction

Maize is an important grain and feed crop. The global requirement for maize continues to grow with the increase in feed and industrial processing demands. In recent years, maize planting has often been threatened by *Fusarium* spp. and *Fusarium* diseases frequently occur in maize-producing areas in China. The major *Fusarium* diseases include corn root rot, sheath rot, stalk rot, and ear rot. *Fusarium* stalk rot and ear rot are the most serious diseases, as they not only reduce maize yield but the toxins produced by *Fusarium* also seriously endanger human and animal health. The *Fusarium* diseases often occur simultaneously.

Corn stalk rot is one of the most destructive corn diseases worldwide, which causes maize lodging and reduces grain weight and yield. Stalk rot typically reduces maize output by 10.0% [1] and may increase to 30.0% to 50.0% in serious cases [2]. The disease was first reported abroad at the beginning of the last century. In the United States and Ontario Canada, it caused yield losses of 349.2 million bushels in 2014 [3]. In China, the disease was widespread in the Northeast region from 2013 to 2015 [4]. In 2014 and 2017, epidemics broke out in the Huang-Huai-Hai region [5,6], and it was serious in 2016 from northern Shanxi Province to western Gansu Province [7]. Stalk rot is caused by many species of *Fusarium* and Pythium. *F. verticillioides*, *F. subglutinans*, and *F. proliferatum* are important fungi that have caused significant losses of maize in the USA [8,9,10,11]. It was reported that corn stalk rot is mainly caused by *F. moniliforme* in Hebei, Hubei, and Guangxi Provinces of China [12], while *F. graminearum* is the main pathogen in Shaanxi, Jilin, and Henan Provinces of China [13]. Pathogen composition is affected by the local environment, and climate conditions and different pathogenic compositions of stalk rot occur in different years in the same area.

Maize ear rot is one of the most prevalent diseases, and the main pathogen is *Fusarium* spp., including *F. verticillioides*, *F. graminearum* species complex (FGSC), *F. oxysporum*, *F. equiseti*, and *F. subglutinans* [14,15,16,17]. Damage from maize ear rot has been on the rise in all maize producing areas in China in recent years. A survey of 10 counties and cities in five provinces of the Huang-Huai-Hai region of China in 2004 showed that the average incidence of ear rot was 42.9% [18]. In 2009, ear rot was serious in all maize growing areas of Gansu Province, with an average ear rate of 63.6% [19]. The occurrence of maize ear rot has also been reported in other countries, among which ear rot in Mexico and Argentina has caused serious losses [20,21]. *Fusarium* spp. produce mycotoxins that are synthesized directly and accumulate in grains; thus, seriously threatening the health of humans and livestock [22,23,24]. The most common *Fusarium* toxins are the fumonisins (FBs), which are mainly produced by *F. verticillioides*. Fumonisin B1 (FB_1_) and fumonisin B2 (FB_2_) are major secondary metabolites of FBs [25]. It has been reported that 50.0% of ear samples are contaminated with FBs in Hebei, Inner Mongolia, Yunnan, Guizhou, Heilongjiang, Liaoning, and Ningxia Provinces [26]. The fumonisins are carcinogenic in humans and livestock and even cause equine leukoencephalomalacia, rat hepatocarcinoma, and porcine pulmonary edema [27,28]. The European Commission (EC) has declared a maximum limit of 4.0 µg/g for unprocessed maize and the US Food and Drug Administration (FDA) has set a maximum level of 2.0 µg/g for maize and maize-based products intended for human consumption [29,30].

Few studies have been conducted on the pathogenic composition between maize ear rot and stalk rot. In this study, the relationship between corn stalk rot and ear rot was analyzed by isolating and identifying *Fusarium* species from stalks, ear stems, and kernels of stalk rot samples. Thus, the present study aimed to provide insight into the relationship between corn stalk rot and ear rot. The study will provide a new perspective for studies on the occurrence pattern of *Fusarium* spp.

## 2. Results

### 2.1. Frequency of Fusarium spp. in Samples

The pathogens were isolated and identified from stalks, ear stems, and corn kernels of 20 samples in 2016 (Table S1). In stalks, the isolation frequencies of *F. equiseti*, *F. verticillioides*, *F. oxysporum*, FGSC, *F. subglutinans*, and *F. proliferatum* were 29.2% (136), 27.1% (126), 21.5% (100), 18.3% (85), 1.9% (9), and 1.1% (5), respectively. *F. equiseti*, *F. verticillioides*, *F. oxysporum*, FGSC, and *F. proliferatum* were isolated from ear stems, with frequencies of 33.3% (31), 28.0% (26), 17.2% (16), 14.0% (13), and 5.4% (5), respectively. In corn kernels, *F. verticillioides*, *F. oxysporum*, *F. equiseti*, FGSC, *F. subglutinans*, and *F. proliferatum* were isolated, with frequencies of 38.0% (228), 36.8% (221), 19.3% (116), 17.8% (107), 3.8% (23), and 0.7% (4), respectively. The main pathogens isolated from stalk, ear stems, and kernels and their isolation frequencies were similar.

Thirteen corn stalk rot samples were collected and the pathogens were identified in 2017. In stalks, *F. verticillioides*, *F. oxysporum*, *F. proliferatum*, FGSC, and *F. equiseti* were isolated, with frequencies of 64.1% (205), 59.7% (191), 8.8% (28), 2.2% (7), and 1.9% (6), respectively. In ear stems, *F. verticillioides*, *F. oxysporum*, *F. equiseti*, FGSC, and *F. proliferatum* were isolated, with frequencies of 69.2% (45), 53.8% (35), 15.4% (10), 10.8% (7), and 6.2% (4), respectively. In corn kernels, *F. verticillioides*, *F. oxysporum*, FGSC, and *F. proliferatum* were isolated, with frequencies of 65.9% (257), 33.9% (87), 18.7% (73), and 10.5% (41), respectively. These results show that the composition of the pathogens isolated from different parts of maize is highly consistent.

The pathogens from 16 samples were isolated and identified in 2018. In stalks, FGSC, *F. verticillioides*, and *F. equiseti* were isolated, with frequencies of 49.5% (193), 24.4% (95), and 2.3% (9), respectively. In ear stems, FGSC, *F. verticillioides*, *F. oxysporum*, *F. equiseti*, *F. proliferatum*, and *F. subglutinans* were isolated, with frequencies of 41.3% (33), 38.8% (31), 10.0% (8), 8.8% (7), 5.0% (4), and 5.0% (4), respectively. In corn kernels, *F. verticillioides*, FGSC, *F. oxysporum*, *F. equiseti*, *F. proliferatum*, and *F. subglutinans* were isolated, with frequencies 38.1% (183), 12.5% (60), 2.1% (10), 1.5% (7), 0.8% (4), and 0.8% (4), respectively. The main pathogens isolated were the same, and *F. verticillioides* was the most important pathogen of kernels.

### 2.2. Fusarium Species in Stems and Ears from Corn Stalk Rot Samples

Based on morphological and molecular identification, a total of 2896 *Fusarium* isolates and six *Fusarium* species were obtained and identified. All specimens were collected from 15 regions in eight provinces from 2016 to 2018. The pathogens isolated from corn kernels, ear stems, and stalks included *F. verticillioides*, *F. oxysporum*, FGSC, *F. equiseti*, *F. proliferatum*, and *F. subglutinans*, with the number of strains isolated of *Fusarium* from different parts were different. The number of isolates of *F. verticillioides* was the highest in all parts (Table 1).

### 2.3. Frequency of Fusarium spp. in Different Parts of Corn

**Figure 1:**
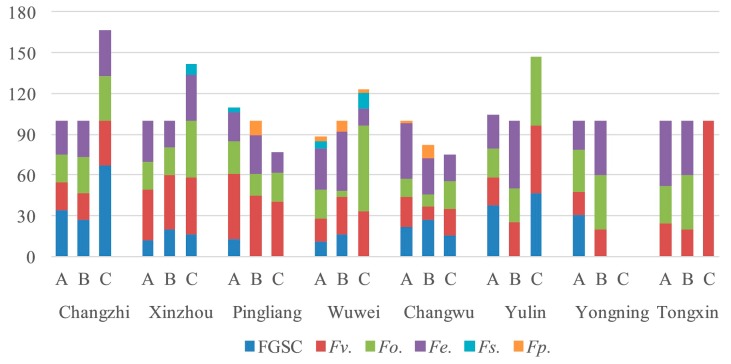
Isolation frequency of *Fusarium* species in different parts of maize in 2016 (A, stalks; B, ear stems; C, kernels).

The *Fusarium* species and their isolation frequency from corn stalks, ear stems, and kernels were consistent from 2016 to 2018. The *Fusarium* species and their isolation frequency in corn stalks, ear stems, and kernels differed in the eight regions in 2016 (Figure 1). All *Fusarium* isolated from corn kernels and ear stems were isolated from stalks in Changzhi, Wuwei, Changwu, Yulin, Yongning, and Tongxin. The main *Fusarium* species from corn kernels and ear stems were isolated from stalks in Xinzhou and Pingliang.

**Figure 2:**
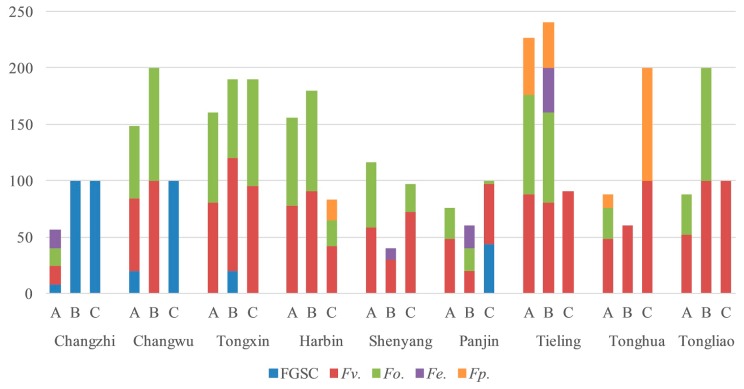
Isolation frequency of *Fusarium* species in different parts of maize in 2017 (A, stalks; B, ear stems; C, kernels).

In 2017, *F. verticillioides* and *F. oxysporum* were isolated from kernels, ear stems, and stalks in Tongxin, Harbin, Shenyang, Tieling, and Tongliao; FGSC was isolated from stalks in Changzhi; and *F. verticillioides* was isolated from stalks in Panjin and Tonghua. In Changwu, the main *Fusarium* isolates from corn kernels and ear stems were isolated from stalks at the same time (Figure 2).

**Figure 3:**
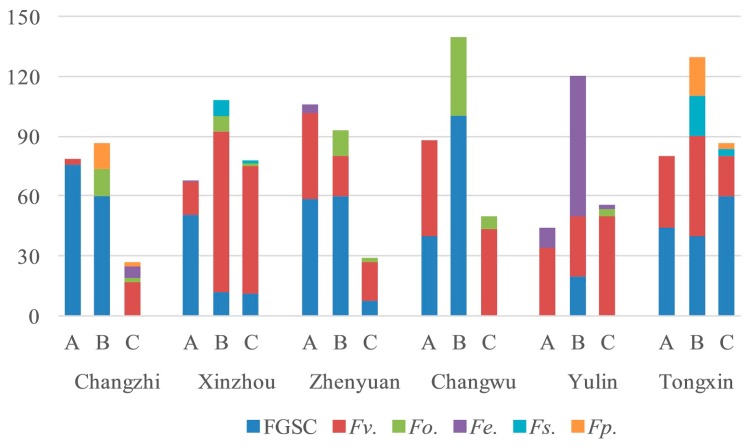
Isolation frequency of *Fusarium* species in different parts of maize in 2018 (A, stalks; B, ear stems; C, kernels).

The same results were observed in Changzhi, Xinzhou, Zhenyuan, Changwu, Yulin, and Tongxin in 2018 (Figure 3). This observation indicates that there is a correlation between stem rot and ear rot.

### 2.4. Fusarium Species in the Different Regions

*F. verticillioides*, *F. oxysporum*, *F. equiseti*, and FGSC were the main pathogens, but there were different isolation frequencies of *Fusarium* spp. in 49 samples in 2016, 2017, and 2018 (Table 2, Table 3 and Table 4). *F. verticillioides* was the main pathogen in samples collected from Pingliang (42.1%), Xinzhou (39.2%), Tongxin (61.7%) in 2016, Harbin (60.8%), Tieling (88.3%), Shenyang (67.5%), Tongxin (85.8%), Panjin (48.3%), Tonghua (75.0%), and Tongliao (80.0%) in 2017, Xinzhou (45.7%), Yulin (41.7%), Changwu (41.7%) in 2018. *F. oxysporum* had a relatively high isolation frequency in the samples from Wuwei (40.3%), Yongning (15.0%) in 2016. FGSC also had a high frequency of isolation from Changzhi (49.4%), Yulin (38.3%) in 2016, Changwu (58.3%), Changzhi (61.7%) in 2017, Zhenyuan (30.0%), Changzhi (38.3%), Tongxin (51.7%) in 2018.

### 2.5. Analysis of Fusarium spp. in Different Maize Varieties

**Figure 4:**
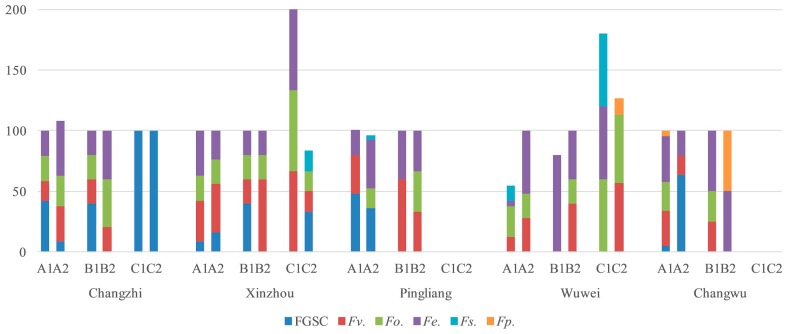
Isolation frequency of *Fusarium* species in different maize varieties in 2016 (A, stalks; B, ear stems; C, kernels; 1, Xianyu 335; 2, Zhengdan 958).

**Figure 5:**
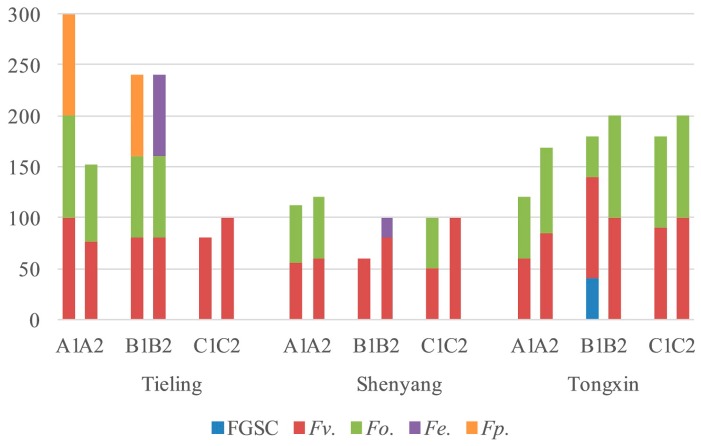
Isolation frequency of *Fusarium* species in different maize varieties in 2017 (A, stalks; B, ear stems; C, kernels; 1, Xianyu 335; 2, Zhengdan 958).

**Figure 6:**
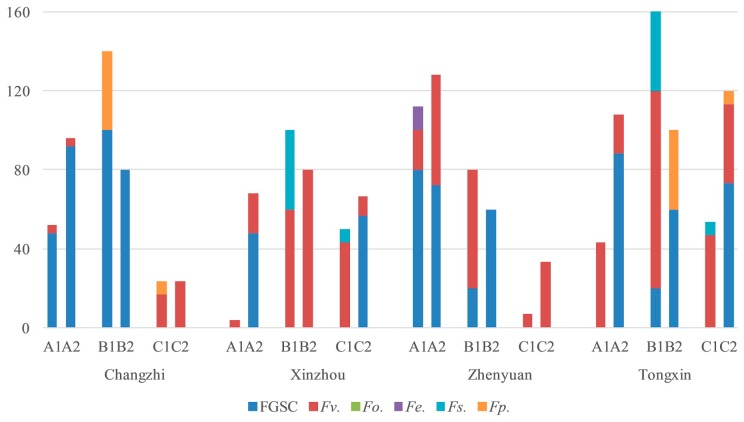
Isolation frequency of *Fusarium* species in different maize varieties in 2018 (A, stalks; B, ear stems; C, kernels; 1, Xianyu 335; 2, Zhengdan 958).

The infection degree and *Fusarium* species of maize varieties are different, it mainly related to geographical location and maize varieties. Zhengdan 958 and Xianyu 335 are the main maize varieties cultured in China. The *Fusarium* contamination rate of Zhengdan 958 was higher in stalks than that of Xianyu 335 in Changzhi, Wuwei, Shenyang, Tongxin, Xinzhou, and Zhenyuan from 2016 to 2018 (Figure 4, Figure 5 and Figure 6). The *Fusarium* infection rate of Zhengdan 958 was the same as that of Xianyu 335 in Xinzhou and Changwu in 2016, while the isolation rate from Zhengdan 958 was lower than that of Xianyu 335 in Pingliang in 2016 and in Tieling in 2017.

The isolation rate of *Fusarium* from ear stems of Zhengdan 958 was higher than that of Xianyu 335 in Wuwei, Shenyang, and Tongxin in 2016 and 2017. The isolation rate of Zhengdan 958 was the same as that of Xianyu 335 in other regions in 2016 and 2017. In 2018, the *Fusarium* contamination rate of Xianyu 335 was higher than that of Zhengdan 958 in Changzhi, Zhenyuan, Xinzhou, and Tongxin.

No *Fusarium* contamination was detected in kernels of either variety from Pingliang or Changwu in 2016. The same isolation rate of *Fusarium* was detected in Zhengdan 958 and Xianyu 335 in Changzhi in 2016, 2018 and Shenyang in 2017. The *Fusarium* infection rate of Zhengdan 958 was lower than that of Xianyu 335 only in Xinzhou and Wuwei in 2016. The proportion of Zhengdan 958 contaminated by *Fusarium* in the remaining five areas (Tieling, Tongxin in 2017; Xinzhou, Zhenyuan, and Tongxin in 2018) was higher than that of Xianyu 335. From the above results, about 67% of the stalks from maize variety Zhengdan 958 were infected by *Fusarium*, which was higher than 16.7% of Xianyu 335.

### 2.6. Analysis of FBs in Kernels

The incidence and levels of fumonisins in maize kernel samples of different regions were tested in 2016, 2017, and 2018 (Table 5, Table 6 and Table 7). A total of 57.1% of all maize samples from 49 samples in 15 regions were contaminated with fumonisins. FBs production in samples was from undetectable (nd) to 5.4 μg/g, 1.1 μg/g, 10.1 μg/g in 2016, 2017, and 2018, respectively. The average concentration of FBs in 2016, 2017, and 2018 were lower than the maximum level (4.0 μg/g) set by the EC. However, some individual samples had higher contamination. The most seriously contaminated area was Yulin (FB_1_ + FB_2_, 10.1 μg/g), followed by Xinzhou (FB_1_ + FB_2_, 6.5 μg/g), Changwu (FB_1_ + FB_2_, 5.4 μg/g), and the main pathogen in these areas was *F. verticillioides*, with an isolation frequency of 100.0%.

**Figure 7:**
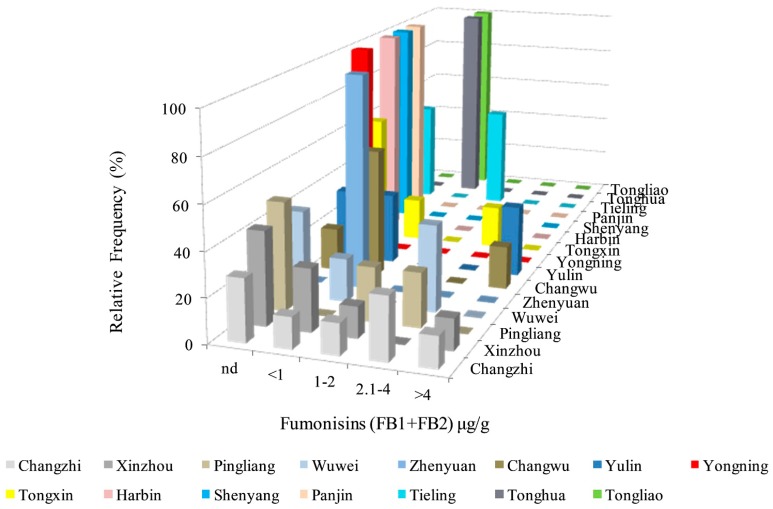
Distribution of fumonisin contamination in maize kernel samples from different regions in 2016, 2017, and 2018 (nd = not detectable).

As shown in Figure 7, different regions had diverse incidences and contamination levels of FBs. About 8.2% of all maize samples were contaminated by more than 4.0 μg/g, as set by the EC, whereas 20.4% of all maize samples were contaminated by more than 2.0 μg/g, as set by the FDA. Yulin (33.3%), Changwu (20.0%), Changzhi (14.3%), and Xinzhou (14.3%) had higher FBs levels than those recommended by the EC. None of the samples from the other 11 regions contained FBs levels above 4.0 μg/g, and 42.9% of all maize samples had nd levels in Changzhi (28.6%), Xinzhou (42.9%), Wuwei (40.0%), Changwu (20.0%), Tongxin (60.0%), Pingliang (50.0%), Yulin (three samples, 33.3%), Harbin (100.0%), Shenyang (100.0%), Tieling (50.0%), Yongning (100.0%), and Panjin (100.0%).

## 3. Discussion

*Fusarium* spp. is a significant pathogen that causes ear rot and stalk rot in maize. *F. verticillioides* and *F. graminearum* are the predominant species causing maize kernel rot in Huang-Huai-Hai and Northeast China [31]. Many studies have focused on the ear rot seed pathogens, but few have reported on the pathogens of ear rot from corn stalk rot samples. Our results indicated that *F. verticillioides*, *F. oxysporum*, and FGSC were the predominant pathogens of maize stalk rot in Northwest China. There were differences in the *Fusarium* species isolated from samples collected from the different regions, mainly related to geographic location and climatic factors during the growing season, such as precipitation, relative humidity, and temperature (Table S2). A relationship has been between the etiology of the *Fusarium* that produces stalk rot and ear rot [32,33,34]. The same kind of *Fusarium* from both diseases can cross-infect. Maize stalk rot aggravates the occurrence of ear rot.

Contamination of kernels with mycotoxin in the field is heavily influenced by multiple factors, such as the pathogen, environmental conditions (temperature, humidity, pH, and light), and host resistance. The production of FBs is closely related to the species of *Fusarium* and the environmental conditions under which it grows. The *Fusarium* species and ability of FBs to affect the host produced by different species varies [35,36]. From 2016 to 2018, 28 of the 49 samples were contaminated with FBs, with a contamination rate of 57.1%. Two maize samples were contaminated by more than 4.0 μg/g, as set by the EC, whereas 10 maize samples were contaminated by more than 2.0 μg/g, as set by the FDA. Areas with a high isolation frequency of *F. verticillioides* do not necessarily have a high level of FBs pollution. In addition to climatic conditions and the ability of strains to produce toxins, the interaction between strains also affects the production of FBs. Resistant varieties should be planted to minimize the risk of FBs to humans and livestock and avoid long-term consumption of corn or corn products with high FBs contents.

**Figure 8:**
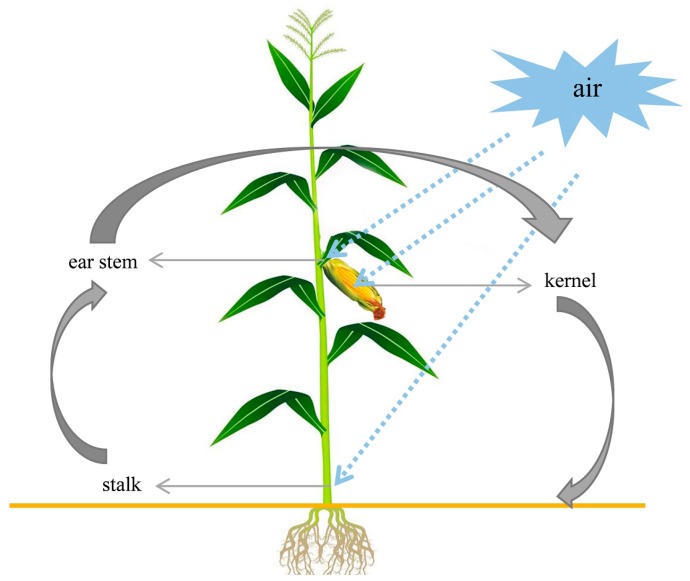
Sampling patterns and pathogen transmission pathways.

Maize stalk rot is a typical transmitted disease occurring during the filling stage of maize. The peak of the disease occurs in the late milk to wax ripening stage. Stem base disease, a hollow interior, and blocked nutrient transport will occur once the disease strikes, seriously limiting yield and 100-grain weight [37]. The disease will affect lodging and a late mechanized harvest [38]. Most stem rot disease infects at the seedling stage, and the pathogen continues to infect throughout the growth period. In severe cases, the pathogen enters the ear of the maize and develops into ear rot [32]. We isolated and identified *Fusarium* spp. from ear stems, stalks, and kernels (Figure 8) and found that the main *Fusarium* isolated from corn kernels and ear stems was isolated from stalks, indicating that the pathogen in kernels caused by a systemic infection in the stalk and pathogens carried in the air may aggravate the occurrence of the diseases. Breeding maize varieties resistant to stem rot is the most economical and effective way to prevent the disease [39]. Zhengdan 958 and Xianyu 335 are the main maize varieties in maize producing areas and are the dominant varieties in production [40]. In stalks, 66.7% of the Zhengdan 958 samples were contaminated with *Fusarium*, which was higher than that of Xianyu 335, but only 25.0% of ear stems and 41.7% of kernels were contaminated (Figure 4, Figure 5 and Figure 6). We will further study the relationship between the maize stalk rot and ear rot to breed disease-resistant varieties.

The pathogen isolation analysis showed a correlation between maize stalk rot and ear rot. It is unknown whether the early invasion by pathogens causes stem rot and ear rot, or whether the nutritional supply is insufficient after stalk rot emerges; plants undergo premature senescence, with a decline in resistance, and ears hang upside down. We plan to detect the spread of pathogens in plants and the relationship between infections of different *Fusarium* species by labeling strains with different colored fluorescence.

## 4. Materials and Methods

### 4.1. Sample Collection and Fungus Isolation and Identification

**Figure 9:**
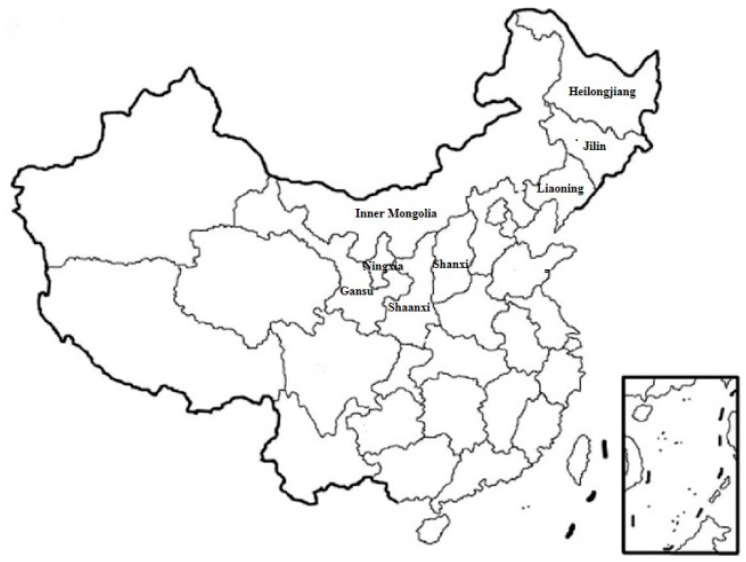
Sampling locations in China.

A total of 49 whole plant corn stalk rot samples representative of corn stalk rot were collected from eight provinces in China from 2016 to 2018 (Figure 9) (Tables S3 and S4). The 30 kernels collected from each sample were soaked in a 1.0% sodium hypochlorite solution for 3 min. Five blocks of ear stems were collected from each sample, and 25 samples from two nodes of stalks near the ground were soaked in 75% anhydrous ethanol, then rinsed three times with sterile water. These kernels and blocks were dried with sterile filter paper and placed on a potato dextrose agar (potato infusion 200.0 g, dextrose 20.0 g, agar 13.0 g, and distilled water 1000 mL) plate for culture for 5 to 6 days at 25 °C. The strain was isolated and identified. The frequency of *Fusarium* isolates was calculated. The frequency of *Fusarium* isolates = number of *Fusarium* strains/number of isolated kernels (or blocks) × 100%.

### 4.2. Identification of Pathogenic Fungi

Genomic DNA was extracted from aerial mycelia using the CTAB method and was validated by species-specific polymerase chain reaction (PCR) (The specific primers were showed in Table 8). Each PCR reaction system (20.0 µL) consisted of a DNA template (2.0 µL), upstream and downstream primers (1.0 µL each), 2× Taq PCR Master Mix (10.0 µL), and double distilled H_2_O (6.0 µL).

The PCR amplification program was initiated at 95 °C for 5 min, followed by 35 cycles at 94 °C for 30 s, annealing for 30 s, annealing temperature according to the primers at 72 °C for 1 min, with a final extension at 72 °C for 10 min. Electrophoretic analysis of the PCR-amplified products was performed on a 1.0% agarose gel.

### 4.3. Detection of Fumonisin FB_1_ and FB_2_

The FBs were analyzed according to a published method [44]. Ten grams of each ground maize sample was randomly and accurately weighed into a 250-mL conical flask and hydrated for 12 h with 10.0 mL ultrapure water. After 12 h, 30.0 mL acetonitrile was added to the mixtures and shaken at 120 r/min for 1 h on a shaking table. The sample extract was filtered through neutral filter paper and collected in a 50-mL plastic centrifuge tube. Eight milliliters of the filtered extract was evaporated and dried. The dried sample residues were re-dissolved in 2.0 mL acetonitrile–water (1:1, *v*/*v*) and filtered through a 0.22 µm nylon filter, before the high-performance liquid chromatography (HPLC) analysis [44,45].

The FB_1_ and FB_2_ contents were detected in this experiment according to a previous method. The incidence and contamination levels of FB_1_ and FB_2_ were analyzed by HPLC coupled with fluorescence detection following o-phthaldialdehyde derivatization [46].

### 4.4. Chemicals and Reagents

The Taq PCR Master Mix, DNA Marker DL2000 were acquired from TransGen Biotech (Beijing, China). Sodium hypochlorite solution, dextrose, agar were purchased from BBI Life Sciences Corporation. FB standards (FB_1_ and FB_2_) were obtained from Sigma Chemical Co. (St. Louis, MO, USA). HPLC-grade acetonitrile and methanol used for sample preparation and mobile phase were supplied by Dikma (Lake Forest, CA, USA). 2-Mercaptoethanol, OPA, and potassium borate buffer o-phthalaldehyde diluent (OD104) were purchased from Pickering Laboratories (Mountain View, CA, USA). Ultrapure water was prepared in all analytical steps using a Barnstead LabTower EDI water purification system (ThermoFisher Scientific, Waltham, MA, USA).

## Figures and Tables

**Table 1:** *Fusarium* species isolated from rotted corn stalks and kernels from 15 regions in China.

*Fusarium* spp.	Number of Strains Isolated			Total
	Corn Kernels	Ear Stems	Stalks	
*F. verticillioides*	668	105	428	1201
*F. oxysporum*	318	59	291	668
FGSC	240	52	282	574
*F. equiseti*	123	44	151	318
*F. proliferatum*	49	13	33	95
*F. subglutinans*	27	4	9	40

**Table 2:** Isolation frequency of *Fusarium* species in different regions in 2016.

Region	No. of Total Samples	Isolation Frequency of *Fusarium* spp.					
		*F. verticillioides*	*F. oxysporum*	*F. proliferatum*	FGSC	*F. equiseti*	*F. subglutinans*
Wuwei	5	25.3%	40.3%	3.3%	5.7%	21.3%	8.0%
Pingliang	4	42.1%	21.7%	0.8%	5.0%	17.9%	1.2%
Changzhi	3	26.7%	27.2%	0.0%	49.4%	28.9%	0.0%
Changwu	3	25.0%	21.7%	1.1%	21.7%	32.2%	0.0%
Xinzhou	2	39.2%	30.8%	0.0%	15.0%	30.8%	4.2%
Yulin	1	35.0%	35.0%	0.0%	38.3%	11.7%	0.0%
Yongning	1	8.3%	15.0%	0.0%	11.7%	11.7%	0.0%
Tongxin	1	61.7%	15.0%	0.0%	0.0%	23.3%	0.0%

**Table 3:** Isolation frequency of *Fusarium* species in different regions in 2017.

Region	No. of Total Samples	Isolation Frequency of *Fusarium* spp.				
		*F. verticillioides*	*F. oxysporum*	*F. proliferatum*	FGSC	*F. equiseti*
Harbin	2	60.8%	51.7%	9.2%	0.0%	0.0%
Tieling	2	88.3%	43.3%	24.2%	0.0%	3.3%
Shenyang	2	67.5%	36.7%	0.0%	0.0%	0.8%
Tongxin	2	85.8%	83.3%	0.0%	1.7%	0.0%
Changwu	1	35.0%	35.0%	0.0%	58.3%	0.0%
Changzhi	1	6.7%	6.7%	0.0%	61.7%	10.0%
Panjin	1	48.3%	15.0%	0.0%	21.7%	8.3%
Tonghua	1	75.0%	11.7%	55.0%	0.0%	0.0%
Tongliao	1	80.0%	23.3%	0.0%	0.0%	0.0%

**Table 4:** Isolation frequency of *Fusarium* species in different regions in 2018.

Region	No. of Total Samples	Isolation Frequency of *Fusarium* spp.					
		*F. verticillioides*	*F. oxysporum*	*F. proliferatum*	FGSC	*F. equiseti*	*F. subglutinans*
Xinzhou	5	45.7%	1.3%	0.0%	28.0%	0.0%	1.3%
Zhenyuan	3	26.7%	2.2%	0.0%	30.0%	1.7%	0.0%
Changzhi	3	9.4%	2.2%	2.2%	38.3%	2.8%	0.0%
Yulin	2	41.7%	1.7%	0.0%	1.7%	11.7%	0.0%
Tongxin	2	29.2%	0.0%	3.3%	51.7%	0.0%	3.3%
Changwu	1	41.7%	6.7%	0.0%	25.0%	0.0%	0.0%

**Table 5:** Incidence and levels of fumonisins in maize kernel samples from different regions in 2016 ^1^.

Region	No. of Total Samples	Fumonisins	No. of Positive Samples (%)	Range (μg/g)	Mean (μg/g)
Changzhi	3	FB_1_	1/3(33.3)	nd–0.3	0.3
		FB_2_	3/3(100.0)	1.7–4.5	2.8
		FB_1_ + FB_2_	3/3(100.0)	1.7–4.5	2.9
Xinzhou	2	FB_1_	0/2(0.0)	nd	-
		FB_2_	1/2(50.0)	nd–1.7	1.7
		FB_1_ + FB_2_	1/2(50.0)	nd–1.73	1.7
Pingliang	4	FB_1_	0/4(0.0)	nd	-
		FB_2_	2/4(50.0)	nd–2.9	2.1
		FB_1_ + FB_2_	2/4(50.0)	nd–2.9	2.1
Wuwei	5	FB_1_	0/5(0.0)	nd	-
		FB_2_	3/5 (60.0)	nd–3.3	2.1
		FB_1_ + FB_2_	3/5 (60.0)	nd–3.3	2.1
Changwu	3	FB_1_	2/3(66.7)	nd–1.2	0.8
		FB_2_	1/3(33.3)	nd–4.3	4.3
		FB_1_ + FB_2_	2/3(66.7)	nd–5.4	2.9
Yulin	1	FB_1_	1/1(100.0)	0.3	0.3
		FB_2_	0/1(0.0)	nd	-
		FB_1_ + FB_2_	1/1(100.0)	0.3	0.3
Tongxin	1	FB_1_	0/1(0.0)	nd	-
		FB_2_	1/1(100.0)	2.5	2.5
		FB_1_ + FB_2_	1/1(100.0)	2.5	2.5
Total	19	FB_1_	4/19(21.1)	nd–1.2	0.6
		FB_2_	11/19(57.9)	nd–4.5	2.5
		FB_1_ + FB_2_	13/19(68.4)	nd–5.4	2.3

^1^ nd = not detected.

**Table 6:** Incidence and levels of fumonisins in maize kernel samples from different regions in 2017 ^1^.

Region	No. of Total Samples	Fumonisins	No. of Positive Samples (%)	Range (μg/g)	Mean (μg/g)
Changwu	1	FB_1_	1/1(100.0)	0.2	0.2
		FB_2_	0/1(0.0)	nd	-
		FB_1_ + FB_2_	1/1(100.0)	0.2	0.2
Changzhi	1	FB_1_	1/1(100.0)	0.1	0.1
		FB_2_	0/1(0.0)	nd	-
		FB_1_ + FB_2_	1/1(100.0)	0.1	0.1
Tieling	2	FB_1_	1/2(50.0)	nd–0.5	0.5
		FB_2_	1/2(50.0)	nd–0.5	0.5
		FB_1_ + FB_2_	1/2(50.0)	nd–1.1	1.1
Tonghua	1	FB_1_	1/1(100.0)	0.1	0.1
		FB_2_	0/1(0.0)	nd	-
		FB_1_ + FB_2_	1/1(100.0)	0.1	0.1
Tongliao	1	FB_1_	1/1(100.0)	0.1	0.1
		FB_2_	0/1(0.0)	nd	-
		FB_1_ + FB_2_	1/1(100.0)	0.1	0.1
Total	6	FB_1_	5/6(83.3)	nd–0.5	0.2
		FB_2_	1/6(16.7)	0.5	0.5
		FB_1_ + FB_2_	5/6(83.3)	nd–1.1	0.3

^1^ nd = not detected.

**Table 7:** Incidence and levels of fumonisins in maize kernel samples from different regions in 2018 ^1^.

Region	No. of Total Samples	Fumonisins	No.of Positive Samples (%)	Range (μg/g)	Mean (μg/g)
Yulin	2	FB_1_	1/2(50.0)	nd–5.4	5.4
		FB_2_	1/2(50.0)	nd–4.7	4.7
		FB_1_ + FB_2_	1/2(50.0)	nd–10.1	10.1
Changwu	1	FB_1_	1/1(100.0)	0.3	0.3
		FB_2_	1/1(100.0)	0.2	0.2
		FB_1_ + FB_2_	1/1(100.0)	0.5	0.5
Changzhi	3	FB_1_	1/3(33.3)	2.7	2.7
		FB_2_	1/3(33.3)	0.7	0.7
		FB_1_ + FB_2_	1/3(33.3)	3.4	3.4
Xinzhou	5	FB_1_	2/5(40.0)	nd–3.8	1.9
		FB_2_	2/5 (40.0)	nd–2.6	1.4
		FB_1_ + FB_2_	3/5 (60.0)	nd–6.5	2.2
Tongxin	2	FB_1_	1/2(50.0)	nd–0.1	0.1
		FB_2_	0/2(0.0)	nd	-
		FB_1_ + FB_2_	1/2(50.0)	nd–0.1	0.1
Total	13	FB_1_	9/13(69.2)	nd–5.4	1.4
		FB_2_	6/13(46.2)	nd–4.7	1.4
		FB_1_ + FB_2_	10/13(76.9)	nd–10.1	2.1

^1^ nd = not detected.

**Table 8:** Specific primer pairs for *Fusarium* spp.

Fungi	Primer	Sequences (5′-3′)	Product Size (bp)	Tm (°C)	Ref.
*F. verticillioides*	VER1 VER2	CTTCCTGCGATGTTTCTCC AATTGGCCATTGGTATTATATATCTA	578	56	[41]
*F. proliferatum*	PRO1 PRO2	CTTTCCGCCAAGTTTCTTC TGTCAGTAACTCGACGTTGTTG	585	56	[41]
*F. subglutinans*	SUB1 SUB2	CTGTCGCTAACCTCTTTATCCA CAGTATGGACGTTGGTATTATATCTAA	631	56	[41]
FGSC	Fg16NF Fg16NR	ACAGATGACAAGATTCAGGCACA TTCTTTGACATCTGTTCAACCCA	280	57	[42]
*F. oxysporum*	FoF1 FoF2	ACATACCACTTGTTGCCTCG CGCCAATCAATTTGAGGAACG	340	58	[43]

## Supplementary Materials

The following are available online at https://www.mdpi.com/2072-6651/11/6/320/s1, Table S1: Isolation frequency of Fusarium species in 49 samples in 2016, 2017, 2018. Table S2: Sampling information. Table S3: Sample collection of corn stalk rot. Table S4: Supplementary samples of corn stalk rot.
