# Supplementary material for: The Relationship Analysis on Corn Stalk Rot and Ear Rot According to Fusarium Species and Fumonisin Contamination in Kernels
Source: Toxins (Basel). 2019 Jun 5;11(6):320. doi: 10.3390/toxins11060320 (PMC6628441; doi:10.3390/toxins11060320)
Supplement: Supplementary file 1 [file toxins-11-00320-s001.pdf]

Supplementary Materials: The Relationship Analysis on Corn Stalk Rot and Ear Rot according to *Fusarium* Species and Fumonisin Contamination in Kernels

Lina Li †, Qing Qu †, Zhiyan Cao *, Zhengyu Guo, Hui Jia, Ning Liu, Yanhui Wang * and Jingao Dong *

† These authors contributed equally to this work.

Table S1. Isolation frequency of *Fusarium* species in 49 samples in 2016, 2017, 2018.

Year	Sample Number	Maize Varieties	Geographic Location	Province	Parts of Maize	*F. verticillioides*	*F. oxysporum*	FGSC	*F. proliferatum*	*F. equiseti*	*F. subglutinas*
2016	1	Zhengdan 958	Changzhi	Shanxi	stalks	29.2%	25.0%	8.3%	0.0%	37.5%	0.0%
					ear stems	20.0%	40.0%	0.0%	0.0%	40%	0.0%
					corn seeds	0.0%	0.0%	100.0%	0.0%	0.0%	0.0%
	2	Luyu 1611	Changzhi	Shanxi	stalks	16.0%	16.0%	52.0%	0.0%	16.0%	0.0%
					ear stems	20.0%	20.0%	40.0%	0.0%	20.0%	0.0%
					corn seeds	100.0%	100.0%	0.0%	0.0%	100.0%	0.0%
	3	Xianyu 335	Changzhi	Shanxi	stalks	16.7%	20.8%	41.7%	0.0%	20.8%	0.0%
					ear stems	20.0%	20.0%	40.0%	0.0%	20.0%	0.0%
					corn seeds	0.0%	0.0%	100.0%	0.0%	0.0%	0.0%
	4	Zhengdan 958	Xinzhou	Shanxi	stalks	40.0%	20.0%	16.0%	0.0%	24.0%	0.0%
					ear stems	60.0%	20.0%	0.0%	0.0%	20.0%	0.0%
					corn seeds	16.7%	16.7%	33.3%	0.0%	0.0%	16.7%
	5	Xianyu 335	Xinzhou	Shanxi	stalks	33.3%	20.8%	8.3%	0.0%	37.5%	0.0%
					ear stems	20.0%	20.0%	40.0%	0.0%	20.0%	0.0%
					corn seeds	66.7%	66.7%	0.0%	0.0%	66.7%	0.0%
	6	Longdan 9	Pingliang	Gansu	stalks	15.8%	10.5%	0.0%	0.0%	26.3%	10.5%
					ear stems	20.0%	0.0%	0.0%	40.0	40.0%	0.0%
					corn seeds	60.0%	86.7%	0.0%	0.0%	60.0%	0.0%
	7	Longdan 10	Pingliang	Gansu	stalks	100.0%	68.0%	0.0%	0.0%	0.0%	0.0%
					ear stems	60.0%	40.0%	0.0%	0.0%	0.0%	0.0%
					corn seeds	100.0%	0.0%	0.0%	0.0%	0.0%	0.0%
	8	Xianyu 335	Pingliang	Gansu	stalks	32.0%	0.0%	48.0%	0.0%	20.0%	0.0%
					ear stems	60.0%	0.0%	0.0%	0.0%	40.0%	0.0%
					corn seeds	0.0%	0.0%	0.0%	0.0%	0.0%	0.0%
	9	Zhengdan 958	Pingliang	Gansu	stalks	36.0%	16.0%	0.0%	0.0%	40.0%	4.0%
					ear stems	33.3%	33.3%	0.0%	0.0%	33.3%	0.0%
					corn seeds	0.0%	0.0%	0.0%	0.0%	0.0%	0.0%
	10	Wuke 609	Wuwei	Gansu	stalks	0.0%	24.0%	28.0%	0.0%	40.0%	8.0%

					ear stems	0.0%	0.0%	80.0%	0.0%	20.0%	0.0%
					corn seeds	46.7%	100.0%	0.0%	0.0%	0.0%	0.0%
	11	Zhengdan 958	Wuwei	Gansu	stalks	28.0%	20.0%	0.0%	0.0%	52.0%	0.0%
					ear stems	40.0%	20.0%	0.0%	0.0%	40.0%	0.0%
					corn seeds	56.7%	56.7%	0.0%	13.3%	0.0%	0.0%
	12	Xianyu 335	Wuwei	Gansu	stalks	12.5%	20.8%	0.0%	0.0%	4.2%	12.5%
					ear stems	0.0%	0.0%	0.0%	0.0%	80.0%	0.0%
					corn seeds	0.0%	60.0%	0.0%	0.0%	60.0%	60.0%
	13	Wuke 620	Wuwei	Gansu	stalks	0.0%	23.8%	4.8%	19.0%	38.1%	0.0%
					ear stems	0.0%	0.0%	0.0%	40.0%	80.0	0.0%
					corn seeds	23.3%	100.0%	0.0%	0.0%	0.0%	0.0%
	14	Wuke 617	Wuwei	Gansu	stalks	42.9%	14.3%	23.8%	0.0%	14.3%	4.8%
					ear stems	100.0%	0.0%	0.0%	0.0%	0.0%	0.0%
					corn seeds	40.0%	0.0%	0.0%	0.0%	0.0%	0.0%
	15	Zhengdan 958	Changwu	Shaanxi	stalks	15.8%	0.0%	63.2%	0.0%	21.1%	0.0%
					ear stems	0.0%	0.0%	0.0%	50.0%	50.0%	0.0%
					corn seeds	100.0%	100.0%	0.0%	0.0%	100.0%	0.0%
	16	Xianyu 335	Changwu	Shaanxi	stalks	28.6%	23.8%	4.8%	4.8%	38.1%	0.0%
					ear stems	50.0%	50.0%	0.0%	0.0%	50.0%	0.0%
					corn seeds	0.0%	0.0%	0.0%	0.0%	0.0%	0.0%
	17	Shandan 609	Changwu	Shaanxi	stalks	23.8%	14.3%	0.0%	0.0%	61.9%	0.0%
					ear stems	0.0%	0.0%	60.0%	0.0%	0.0%	0.0%
					corn seeds	0.0%	0.0%	76.7%	0.0%	0.0%	0.0%
	18	Zhengdan 958	Yulin	Shaanxi	stalks	20.8%	20.8%	37.5%	0.0%	25.0%	0.0%
					ear stems	25.0%	25.0%	0.0%	0.0%	80.0%	0.0%
					corn seeds	50.0%	50.0%	46.7%	0.0%	0.0%	0.0%
	19	Longdan 9	Yongning	Ningxia	stalks	17.4%	30.4%	30.4%	0.0%	21.7%	0.0%
					ear stems	20.0%	40.0%	0.0%	0.0%	40.0%	0.0%
					corn seeds	0.0%	0.0%	0.0%	0.0%	0.0%	0.0%
	20	Xianyu 335	Tongxin	Ningxia	stalks	24.0%	28.0%	0.0%	0.0%	48.0%	0.0%
					ear stems	20.0%	40.0%	0.0%	0.0%	40.0%	0.0%
					corn seeds	0.0%	0.0%	0.0%	0.0%	0.0%	0.0%
2017	21	Zhengdan 958	Harbin	Heilongjiang	stalks	96.0%	96.0%	0.0%	0.0%	0.0%	0.0%
					ear stems	100.0%	100.0%	0.0%	0.0%	0.0%	0.0%
					corn seeds	36.7%	0.0%	0.0%	0.0%	0.0%	0.0%
	22	Keyu 16	Harbin	Heilongjiang	stalks	60.0%	60.0%	0.0%	0.0%	0.0%	0.0%
					ear stems	80.0%	80.0%	0.0%	0.0%	0.0%	0.0%
					corn seeds	46.7%	46.7%	0.0%	36.7%	0.0%	0.0%
	23	Xianyu 335	Changwu	Shaanxi	stalks	64.0%	64.0%	20.0%	0.0%	0.0%	0.0%
					ear stems	100.0%	100.0%	0.0%	0.0%	0.0%	0.0%

					corn seeds	0.0%	0.0%	100.0%	0.0%	0.0%	0.0%
					stalks	16.0%	16.0%	8.0%	0.0%	24.0%	0.0%
	24	Xianyu 696	Changzhi	Shanxi	ear stems	0.0%	0.0%	100.0%	0.0%	0.0%	0.0%
					corn seeds	0.0%	0.0%	100.0%	0.0%	0.0%	0.0%
					stalks	76.0%	76.0%	0.0%	0.0%	0.0%	0.0%
	25	Zhengdan 958	Tieling	Liaoning	ear stems	80.0%	80.0%	0.0%	0.0%	80.0%	0.0%
					corn seeds	100.0%	0.0%	0.0%	0.0%	0.0%	0.0%
					stalks	100.0%	100.0%	0.0%	100.0%	0.0%	0.0%
	26	Xianyu 335	Tieling	Liaoning	ear stems	80.0%	80.0%	0.0%	80.0%	0.0%	0.0%
					corn seeds	80.0%	0.0%	0.0%	0.0%	0.0%	0.0%
					stalks	56.0 %	56.0%	0.0%	0.0%	0.0%	0.0%
	27	Xianyu 335	Shenyang	Liaoning	ear stems	60.0%	0.0%	0.0%	0.0%	0.0%	0.0%
					corn seeds	50.0%	50.0%	0.0%	0.0%	0.0%	0.0%
					stalks	48.0%	28.0%	0.0%	0.0%	0.0%	0.0%
	28	Zhengdan 958	Panjin	Liaoning	ear stems	20.0%	20.0%	0.0%	0.0%	100.0%	0.0%
					corn seeds	53.3%	3.3%	43.3%	0.0%	0.0%	0.0%
					stalks	60.0%	60.0%	0.0%	0.0%	0.0%	0.0%
	29	Zhengdan 958	Shenyang	Liaoning	ear stems	0.0%	0.0%	0.0%	0.0%	20.0%	0.0%
					corn seeds	100.0%	0.0%	0.0%	0.0%	0.0%	0.0%
					stalks	48.0%	28.0%	0.0%	12.0%	0.0%	0.0%
	30	Xianyu 335	Tonghua	Jilin	ear stems	60.0%	0.0%	0.0%	0.0%	0.0%	0.0%
					corn seeds	100.0%	0.0%	0.0%	100.0%	0.0%	0.0%
					stalks	52.0%	36.0%	0.0%	0.0%	0.0%	0.0%
	31	Qingchu 368	Tongliao	Inner Mongolia	ear stems	100.0%	100.0%	0.0%	0.0%	0.0%	0.0%
					corn seeds	100.0%	0.0%	0.0%	0.0%	0.0%	0.0%
					stalks	84.0%	84.0%	0.0%	0.0%	0.0%	0.0%
	32	Zhengdan 958	Tongxin	Ningxia	ear stems	100.0%	100.0%	0.0%	0.0%	0.0%	0.0%
					corn seeds	100.0%	100.0%	0.0%	0.0%	0.0%	0.0%
					stalks	60.0%	60.0%	0.0%	0.0%	0.0%	0.0%
	33	Xianyu 335	Tongxin	Ningxia	ear stems	100.0%	40.0%	40.0%	0.0%	0.0%	0.0%
					corn seeds	90.0%	90.0%	0.0%	0.0%	0.0%	0.0%
					stalks	36.0%	0.0%	0.0%	0.0%	0.0%	0.0%
	34	Shandan 609	Yulin	Shaanxi	ear stems	60.0%	0.0%	20.0%	0.0%	40.0%	0.0%
					corn seeds	0.0%	6.7%	0.0%	0.0%	6.7%	0.0%
					stalks	32.0%	0.0%	0.0%	0.0%	20.0%	0.0%
2018	35	Zhengdan 958	Yulin	Shaanxi	ear stems	0.0%	0.0%	20.0%	0.0%	100.0%	0.0%
					corn seeds	100.0%	0.0%	0.0%	0.0%	0.0%	0.0%
					stalks	48.0%	40.0%	0.0%	0.0%	0.0%	0.0%
	36	Xianyu 335	Changwu	Shaanxi	ear stems	0.0%	40.0%	100.0%	0.0%	0.0%	0.0%
					corn seeds	43.3%	6.7%	0.0%	0.0%	0.0%	0.0%

				stalks	4.0%	0.0%	48.0%	0.0%	0.0%	0.0%
37	Xianyu 335	Changzhi	Shanxi	ear stems	0.0%	0.0%	100.0%	40.0%	0.0%	0.0%
				corn seeds	16.7%	0.0%	0.0%	6.7%	0.0%	0.0%
				stalks	4.0%	0.0%	92.0%	0.0%	0.0%	0.0%
38	Zhengdan 958	Changzhi	Shanxi	ear stems	0.0%	0.0%	80.0%	0.0%	0.0%	0.0%
				corn seeds	23.3%	0.0%	0.0%	0.0%	0.0%	0.0%
				stalks	0.0%	0.0%	100.0%	0.0%	0.0%	0.0%
39	Dika 517	Changzhi	Shanxi	ear stems	0.0%	40.0%	0.0%	0.0%	0.0%	0.0%
				corn seeds	10.0%	6.7%	0.0%	0.0%	16.7%	0.0%
				stalks	4.0%	0.0%	0.0%	0.0%	0.0%	0.0%
40	Xianyu 335	Xinzhou	Shanxi	ear stems	60.0%	0.0%	0.0%	0.0%	0.0%	40.0%
				corn seeds	43.3%	0.0%	0.0%	0.0%	0.0%	6.7%
				stalks	20.0%	0.0%	48.0%	0.0%	0.0%	0.0%
41	Zhengdan 958	Xinzhou	Shanxi	ear stems	80.0%	0.0%	0.0%	0.0%	0.0%	0.0%
				corn seeds	10.0%	0.0%	56.7%	0.0%	0.0%	0.0%
				stalks	16.0%	0.0%	52.0%	0.0%	4.0%	0.0%
42	M753	Xinzhou	Shanxi	ear stems	60.0%	0.0%	40.0%	0.0%	0.0%	0.0%
				corn seeds	90.0%	0.0%	0.0%	0.0%	0.0%	0.0%
				stalks	40.0%	0.0%	88.0%	0.0%	0.0%	0.0%
43	Dafeng 30	Xinzhou	Shanxi	ear stems	100.0%	40.0%	0.0%	0.0%	0.0%	0.0%
				corn seeds	100.0%	6.7%	0.0%	0.0%	0.0%	0.0%
				stalks	4.0%	0.0%	68.0%	0.0%	0.0%	0.0%
44	Yufeng 303	Xinzhou	Shanxi	ear stems	100.0%	0.0%	20.0%	0.0%	0.0%	0.0%
				corn seeds	76.7%	0.0%	0.0%	0.0%	0.0%	0.0%
				stalks	20.0%	0.0%	88.0%	0.0%	0.0%	0.0%
45	Zhengdan 958	Tongxin	Ningxia	ear stems	0.0%	0.0%	60.0%	40.0%	0.0%	0.0%
				corn seeds	40.0%	0.0%	73.3%	6.7%	0.0%	0.0%
				stalks	52.0%	0.0%	0.0%	0.0%	0.0%	0.0%
46	Xianyu 335	Tongxin	Ningxia	ear stems	100.0%	0.0%	20.0%	0.0%	0.0%	40.0%
				corn seeds	0.0%	0.0%	46.7%	0.0%	0.0%	6.7%
				stalks	56.0%	0.0%	72.0%	0.0%	0.0%	0.0%
47	Zhengdan 958	Zhenyuan	Gansu	ear stems	0.0%	0.0%	60.0%	0.0%	0.0%	0.0%
				corn seeds	33.3%	0.0%	0.0%	0.0%	0.0%	0.0%
				stalks	20.0%	0.0%	80.0%	0.0%	12.0%	0.0%
48	Xianyu 335	Zhenyuan	Gansu	ear stems	60.0%	0.0%	20.0%	0.0%	0.0%	0.0%
				corn seeds	6.7%	0.0%	0.0%	0.0%	0.0%	0.0%
				stalks	60.0%	0.0%	0.0%	0.0%	0.0%	0.0%
49	Yuyu 22	Zhenyuan	Gansu	ear stems	0.0%	40.0%	100.0%	0.0%	0.0%	0.0%
				corn seeds	16.7%	6.7%	23.3%	0.0%	0.0%	0.0%

Table S2. Sampling information.

Region	No. of Total Samples	Average Temperature (°C)	Mean Relative Humidity (%)	Sample Year
Wuwei	5	25	38	2016
Pingliang	4	23	60	2016
Changzhi	3	25	58	2016
Changwu	3	22	47	2016
Xinzhou	2	25	55	2016
Yulin	1	23	44	2016
Yongning	1	27	39	2016
Tongxin	1	26	40	2016
Harbin	2	21	63	2017
Tieling	2	24	67	2017
Shenyang	2	24	60	2017
Tongxin	2	24	38	2017
Changwu	1	20	55	2017
Changzhi	1	24	55	2017
Panjin	1	25	66	2017
Tonghua	1	23	72	2017
Tongliao	1	24	51	2017
Xinzhou	5	22	54	2018
Zhenyuan	3	21	57	2018
Changzhi	3	21	57	2018
Yulin	2	19	45	2018
Tongxin	2	21	42	2018
Changwu	1	20	45	2018

Table S3. Sample collection of corn stalk rot.

Province	Geographic Location	Year	Zhengdan 958	Xianyu 335	Local Main Varieties
Shanxi	Changzhi	2016	√	√	Luyu 1611
		2017			Xianyu 696
		2018	√	√	Dika 517
	Xinzhou	2016	√	√	
		2018	√	√	M753<br>Dafeng30<br>Yufeng303
Shaanxi	Changwu	2016	√	√	Shandan 609
		2017		√	
		2018		√	
	Yulin	2016	√		
		2018	√		Shandan 609
Ningxia	Tongxin	2016		√	
		2017	√	√	
		2018	√	√	

Table S4. Supplementary samples of corn stalk rot.

Province	Geographic Location	Year	Zhengdan 958	Xianyu 335	Local Main Varieties
Gansu	Pingliang	2016	√	√	Longdan 9<br>Longdan 10
	Wuwei	2016	√	√	Wuke 609<br>Wuke 620<br>Wuke 617
	Zhenyuan	2018	√	√	Yuyu 22
Ningxia	Yongning	2016			Longdan 9
Heilongjiang	Harbin	2017	√		Keyu 16
Liaoning	Tieling	2017	√	√	
	Shenyang	2017	√	√	
	Panjin	2017	√		
Jilin	Tonghua	2017		√	
Inner Mongolia	Tongliao	2017			Qingchu 368
